# 

**Published:** 1899-02-18

**Authors:** 


**Notices of Births, Deaths, and Marriages** are inserted in this column at a charge of 2s. 6d. for an announcement not exceeding four lines.

### NOTICE TO CORRESPONDENTS.

All MS., Letters, Books for Review, and other matters intended for the Editor should be addressed The Editor, "The Hospital," The Hospital Building, 28 & 29, Southampton Street, Strand, W.C.

The Editor cannot undertake to return rejected MS., even when accompanied by stamped directed envelope.

**Notice to Advertisers and Subscribers.**

All Advertisements, Orders for Copies of The Hospital, and Business Communications should be addressed to **THE MANAGER (Not to the Editor),** The Hospital, The Hospital Building, 28 & 29, Southampton Street, Strand, London, W.C.

The Cost of Subscription, post free, is as follows:—Per week, 2½d.; per quarter, 2s. 8d.; per half-year, 5s. 3d.; per annum, 10s. 6d. Foreign:—Per annum, 15s. 2d., payable, in all cases, in advance.

**NOTICE.—Vol. XXIV. of "The Hospital" (April, 1898, to September, 1898), in handsome cloth binding, is now on sale at the Office of this Journal, price 7s. 6d. Cases for binding the Numbers contained in this Volume 1s. 6d. Index to Vol. XXIV., 2½d., post free.**

**The Monthly Part for March will be ready March 1st. Price 6d., by post 8d.**

### SCRAPS AND GLEANINGS.

Three of the District Councils concerned have refused to join the Swansea Corporation in a joint isolation hospital.

The Birmingham Hospital Sunday collections this year on behalf of the Birmingham General Hospital have amounted to £5,246 odd, which is an increase of £824 over the amount collected in 1895.

The expenditure at the Coventry and Warwickshire Hospital for December, 1898, was £301 7s. 8d., as against £367 8s. 9d. for the same period last year, thus showing a decrease of £66, notwithstanding that there was an increase of two in the number of patients.

The sub-committee appointed to inquire into the accommodation for the sick and other matters at the Romsey Workhouse Infirmary have reported that, in their opinion, an enlargement of the infirmary is absolutely necessary. The Board have sanctioned the inviting of the medical officer and the architect to confer with the committee.

A circular has been issued by the committee of the New Infirmary Fund, Newcastle, informing the subscribers that the scheme for the erection of an infirmary with 400 beds has been adopted; that the cost, according to Mr. Waterhouse's estimate, will be at least £160,000; and that, after accepting Mr. John Hall's generous offer for building and furnishing, it is proposed to provide the further sum required out of the Jubilee Fund raised by public subscription, the surplus being invested as an endowment to assist in carrying on the infirmary.

Crown 8vo., Paper Covers, 1s.,

**SOME HEALTH ASPECTS OF EDUCATION.**

By PERCY G. LEWIS, M.D., M.R.C.S., L.S.A.,

Author of "Nursing: Its Theory and Practice."

"A decidedly practical pamphlet, which has for its object the direction of attention to the possible sanitary defects of a child, and the principles to be observed in repairing them."—*School Guardian.*

London: The Scientific Press, Ltd., 28 & 29, Southampton Street, Strand, W.C.

